# The alternations of gut microbiota in diabetic kidney disease: insights from a triple comparative cohort

**DOI:** 10.3389/fcimb.2025.1606700

**Published:** 2025-06-27

**Authors:** Mengqi Wu, Xin Zhou, Saiping Chen, Yuqing Wang, Bin Lu, Aiping Zhang, Yanqin Zhu, Min Huang, Jiarui Wang, Junyi Liu, Fenggui Zhu, Hong Liu, Riyang Lin

**Affiliations:** ^1^ Department of Nephrology, Hangzhou Traditional Chinese Medicine Hospital of Zhejiang Chinese Medical University, Hangzhou, Zhejiang, China; ^2^ Department of General Medicine, Tianshui Wulin Street Community Heal Care Centre, Hangzhou, Zhejiang, China; ^3^ Key Laboratory of Kidney Disease Prevention and Control Technology, Hangzhou, Zhejiang, China

**Keywords:** diabetic kidney disease (DKD), gut microbiota, 16S rRNA, intestinal biomarker, disease progression

## Abstract

**Background:**

Diabetic kidney disease (DKD) exhibits heterogeneous progression, implicating factors beyond hyperglycemia, such as gut microbiota dysbiosis. However, microbial distinctions among biopsy-confirmed pure DKD, DKD with non-diabetic renal disease (DKD+NDRD), and long-term diabetes without nephropathy (DM) remain unclear. This study aimed to identify gut microbial and functional biomarkers differentiating these groups.

**Methods:**

We enrolled 40 biopsy-confirmed participants classified into DKD (n=26), DM (n=8), and DKD+NDRD (n=6) groups. Gut microbiota was profiled using 16S rRNA sequencing. Microbial diversity, composition, and functional prediction (PICRUSt2 analysis) were compared among groups. Biomarkers were identified using LEfSe analysis.

**Results:**

No significant differences in alpha-diversity (Chao1, Shannon indices) or beta-diversity (PCoA/PCA) were observed among groups. Taxonomic analysis revealed distinct microbial signatures: DKD patients showed enrichment of Olsenella and reduced Faecalibacterium prausnitzii (a short-chain fatty acid producer), while DM patients exhibited higher Roseburia and Flavonifractor. The DKD+NDRD group was uniquely enriched in Prevotella_9. Functional prediction highlighted elevated pyruvate metabolism and bacterial toxin pathways in DKD, contrasting with enhanced linoleic acid metabolism in DM and attenuated endotoxin-related pathways in DKD+NDRD.

**Conclusions:**

This study delineates gut microbiota profiles and functional shifts across DKD, DM, and DKD+NDRD. Key taxa (Olsenella, Prevotella_9) and metabolic pathways (pyruvate, toxin production) may serve as biomarkers for DKD progression and differential diagnosis. The findings underscore the gut-kidney axis’s role in DKD pathogenesis and suggest microbiota-targeted interventions for precision management. Further validation in larger cohorts is warranted.

## 1 Introduction

Diabetic kidney disease (DKD) represents a major microvascular complication of diabetes, affecting around 40% of patients globally and frequently progressing to end-stage renal disease (ESRD) (Thomas et al., 2015; Alicic et al., 2017). Although hyperglycemia is central to DKD pathogenesis, its progression varies significantly among individuals: 20-30% of T1DM patients develop microalbuminuria within 5-10 years, and about 20% of T2DM patients show microalbuminuria at diagnosis (Papadopoulou-Marketou et al., 2018), whereas others maintain preserved renal function even after long-standing disease. Such heterogeneity implies that factors beyond dysglycemia, including genetics and gut microbiota alterations, may play a role in DKD development and progression.

In recent decades, gut microbiota imbalance and its mechanistic links to diseases including diabetes, CKD, inflammatory bowel disease, dyslipidemia, obesity, and cardiovascular conditions have attracted increasing research attention (Lau and Vaziri, 2019). Evidence from animal studies indicates that renal dysfunction in rats is strongly linked to reduced levels of fecal probiotics, particularly Lactobacillus and Bifidobacterium (Miao et al., 2024b). This microbial shift may reflect early gut-kidney axis alterations that contribute to disease progression. A number of seminal reviews have revealed multiple host-derived uremic toxins that promote the advancement of CKD (Mafra et al., 2021; Miao et al., 2024a); these reviews also highlighted that CKD patients exhibit elevated levels of Fusobacteria, Proteobacteria, Streptococcus, Escherichia_Shigella, and Desulfovibrionota, alongside significant reductions in Faecalibacterium, Prevotella_9 (a genus-level clade classified under the Prevotella genus in the SILVA 16S database), and Roseburia. There is a documented reduction in L. johnsonii, L. murinus, L. vaginalis, L. reuteri, and B. animalis (Meijers et al., 2019; Zhao et al., 2021; Krukowski et al., 2023), which coincides with elevated concentrations of indoxyl sulfate and p-cresyl sulfate as well as a decline in short-chain fatty acid (SCFA) production (Meijers et al., 2019; Koppe and Soulage, 2022). Collectively, these findings suggest a consistent pattern of dysbiosis in CKD that may influence systemic toxicity and inflammation.

A comprehensive bibliometric study identified two major mechanisms underlying gut–kidney axis impairment (Tao et al., 2024). Firstly, the progression of CKD is commonly associated with disrupted gut microbiota, marked by a loss of beneficial bacteria and a rise in pathogenic species. Such microbial imbalance facilitates the buildup of uremic toxins in circulation, leading to progressive renal dysfunction (Chen et al., 2019; Cosola et al., 2019). Secondly, dysbiosis of the gut microbiota disrupts the intestinal barrier, enabling translocation of uremic toxins and microbial pathogens into systemic circulation. The ensuing immune activation in the gut mucosa induces low-grade systemic inflammation, thereby worsening kidney damage (Rochus et al., 2014; Khoury et al., 2017). Together, these findings underscore the pivotal role of gut dysbiosis in the pathophysiology of CKD. The maintenance of renal health is intimately associated with the homeostasis of gut microbial communities and their metabolic products. Microbial imbalance and the presence of dysbiosis-associated metabolites have been implicated in the pathogenesis of kidney damage and renal fibrosis (Giordano et al., 2021; Tian et al., 2023; Wang et al., 2023). These insights support the rationale for developing microbiota-based interventions. Theoretically, targeted restoration of gut–kidney axis homeostasis offers a compelling approach to CKD treatment (Li et al., 2024). Recent studies have shown that a higher dietary index for gut microbiota (DI-GM) scores is associated with a lower prevalence of CKD (OR=0.958) (Xiao et al., 2025). Furthermore, natural products such as resveratrol, curcumin, and emodin exert therapeutic benefits in CKD through gut microbiota modulation, with notable shifts in Lactobacillus, Akkermansia, and Bacteroides levels (Li et al., 2024). Scholars have further advocated for targeted therapies aimed specifically at correcting microbial dysbiosis. For instance, Miao H et al. demonstrated that L. johnsonii therapy increases serum IAld concentrations, suppresses AHR signaling, and consequently attenuates renal pathology in CKD (Miao et al., 2024a). This provides a mechanistic example of how probiotic interventions can translate into molecular and clinical benefits.

In diabetes, microbiota imbalance impairs the intestinal barrier, enabling microbial-derived uremic toxins to circulate systemically and induce inflammation, oxidative stress, insulin resistance, β-cell dysfunction, and kidney damage (Koppe et al., 2018). Prior research has indicated that gut microbiota plays a central regulatory role in DKD development among individuals with diabetes (Andrade-Oliveira et al., 2015; Tao et al., 2019). Gut microbiota dysbiosis in DKD patients promotes various cellular processes, such as the citrate cycle, base excision and repair, histidine metabolism, lipoic acid metabolism, and bile acid biosynthesis. These altered pathways, in turn, influence host glucose metabolism and immune-inflammatory responses via multiple signaling cascades, notably the MAPK/NF-κB pathway, thereby exacerbating renal damage in DKD patients (Chu et al., 2024; Xu et al., 2024; Cao et al., 2025). However, many DKD patients may also present with overlapping non-diabetic renal disease (NDRD), and the lack of strict differentiation in existing studies may obscure the detection of DKD-specific microbial signatures.

To address this gap, this study employed a novel triple-control cohort design: (1) biopsy-confirmed DKD without NDRD; (2) DM with disease duration >10 years and preserved renal function; (3) DKD+NDRD, confirmed by pathology. By integrating 16S rDNA sequencing with metabolic profiling, we comprehensively analyzed DKD-specific microbial signatures and functions. Our findings may not only help identify early microbial markers of DKD but also lay the foundation for precision interventions targeting the gut–kidney axis.

## 2 Methods

### 2.1 Study participants

Based on standardized diagnostic, inclusion, and exclusion criteria, patients visiting the Department of Endocrinology and Nephrology of Hangzhou Hospital of Traditional Chinese Medicine between June and December 2023 were enrolled. According to the ADA diagnostic criteria for T2DM (2022) and the DKD pathology guidelines from Renal Biopsy Pathology (Zou, 2016), participants were divided into three groups: DKD, DM, and DKD with NDRD (DKD+NDRD). All diagnoses were confirmed through clinical records and pathological assessments. Stool specimens were randomly collected from 40 patients: 26 from the DKD group, 8 from the DM group, and 6 from the DKD+NDRD group. Specimens were collected before any antibiotic or probiotic exposure to minimize external microbial interference.

The DKD group met the following inclusion criteria:

1. Aged 18–75 years;
2. Diagnosed with type 2 diabetes;
3. Pathological confirmation of DKD via renal biopsy;
4. No evidence of primary renal diseases (e.g., primary glomerulonephritis, IgA nephropathy, lupus nephritis) as determined by pathology and clinical findings;
5. Ability to understand the study and provide written informed consent.

The DM group met the following inclusion criteria:

1. Aged 18–75 years;
2. History of type 2 diabetes mellitus for more than 10 years;
3. No microvascular complications, such as diabetic retinopathy or nephropathy, with eGFR ≥ 60 ml/min/1.73 m²;
4. Ability to understand the study and provide written informed consent.

The DKD+NDRD group met the following criteria:

1. Aged 18–75 years;
2. Diagnosed with type 2 diabetes;
3. Renal biopsy showing pathological features of DKD along with features of other NDRD;
4. Ability to understand the study and provide written informed consent.

Exclusion criteria:

1. Patients with malignancies, including solid tumors and hematologic cancers;
2. Patients with severe liver dysfunction (ALT or AST >3× upper limit of normal, or diagnosed cirrhosis);
3. Pregnant or breastfeeding women;
4. Those receiving renal replacement therapy, including hemodialysis, peritoneal dialysis, or kidney transplantation;
5. Patients with serious cardiovascular diseases such as acute myocardial infarction or NYHA class III–IV heart failure;
6. History of gastrointestinal infection within the past month, including acute gastroenteritis, Helicobacter pylori infection, or other bacterial/viral intestinal infections;
7. Use of antibiotics within the past month;
8. Incomplete baseline data.

All procedures performed in this study involving human participants were conducted in accordance with ethical standards and were approved by the Biomedical Ethics Committee of Hangzhou Hospital of Traditional Chinese Medicine. Written informed consent was obtained from all patients or participants.

### 2.2 Fecal sample collection and DNA extraction

Fresh stool samples were obtained from the enrolled participants. All samples were collected on the same day as renal biopsy, prior to the initiation of any therapeutic intervention. Stool specimens were placed into sterile containers, immediately transported on dry ice to the laboratory within 2 hours post-collection, and stored at −80°C until further processing. Microbial genomic DNA was extracted using the CTAB method, and DNA integrity and concentration were evaluated via agarose gel electrophoresis. Briefly, 0.25 g of stool sample was suspended in CTAB buffer, followed by incubation with lysozyme and proteinase K at 37°C for 1 hour. DNA was extracted using isopropanol precipitation, washed with ethanol, and resuspended in sterile water. DNA quality was assessed using 1% agarose gel electrophoresis and Nanodrop 2000 spectrophotometry (Thermo Scientific).

### 2.3 16S rRNA gene amplification and sequencing

Polymerase Chain Reaction (PCR) amplification was performed using the forward primer 338F (5’-ACTCCTACGGGAGGCAGCAG-3’) and the reverse primer 806R (5’-GGACTACHVGGGTWTCTAAT-3’). The PCR reactions were conducted in 30 μL volumes containing 15 μL Phusion^®^ High-Fidelity PCR Master Mix (New England Biolabs), 0.2 μM of each primer, and 10 ng template DNA. The cycling conditions were: initial denaturation at 98°C for 1 minute; 30 cycles of 98°C for 10 seconds, 50°C for 30 seconds, 72°C for 30 seconds; and a final extension at 72°C for 5 minutes. The PCR products were purified using AMPure XT beads (Beckman Coulter Genomics, Danvers, MA, USA) and quantified with a Qubit fluorometer (Invitrogen, USA). The PCR amplicons were verified by 2% agarose gel electrophoresis. The purified PCR products were further evaluated using the Agilent 2100 Bioanalyzer (Agilent, USA) and quantified with the Illumina library quantification kit (Kapa Biosciences, Woburn, MA, USA). Only libraries with a concentration above 2 nM were considered acceptable. Qualified sequencing libraries (with non-redundant index sequences) were diluted in a gradient manner, mixed proportionally according to the required sequencing volume, and denatured with NaOH to generate single-stranded DNA for sequencing. Paired-end sequencing (2×250 bp) was performed using the NovaSeq 6000 Sequencer with the NovaSeq 6000 SP Reagent Kit (500 cycles). All sequencing was conducted by Lianchuan BioTech (Hangzhou, China).

### 2.4 Statistical analysis

Amplicon sequence variants (ASVs) were annotated using the SILVA and NT-16S databases. The abundance of each taxon was calculated based on the ASV abundance table. Subsequently, microbial diversity was assessed through both α-diversity and β-diversity analyses, using the ASV feature sequences and abundance tables. α-diversity was used to evaluate within- and between-group richness and evenness. Metrics used included observed species, Shannon index, Simpson index, Chao1 index, and Pielou’s evenness. β-diversity was evaluated using weighted UniFrac distances and visualized through principal coordinate analysis (PCoA) and principal component analysis (PCA). Differential taxa were identified using Linear Discriminant Analysis Effect Size (LEfSe) to screen for statistically and biologically relevant biomarkers. The Mann–Whitney U test was used for pairwise comparisons, and the Kruskal–Wallis test was applied for comparisons across multiple groups. P-values were adjusted using the Benjamini–Hochberg FDR method, with p < 0.05 considered significant. Functional prediction was performed using PICRUSt2 (v2.2.0b) to infer potential metagenomic functions from 16S ASV data. The ASV table and representative sequences were aligned with reference phylogenies using an NSTI threshold of 2. For each ASV, functional gene families and copy numbers were predicted. Final pathway annotations were derived using the KEGG database and analyzed in STAMP using t-tests. Given the limited sample size and exploratory study design, additional multivariate analyses such as PERMANOVA or adjusted regression modeling were not performed. This is acknowledged as a limitation and will be addressed in future studies with larger cohorts.

## 3 Results

### 3.1 General characteristics of all participants

We enrolled 26 biopsy-confirmed DKD patients with a median age of 59, 6 patients diagnosed with DKD combined with NDRD (DKD+NDRD) with a median age of 56.5, and 8 long-duration DM patients without renal involvement. Baseline demographic and clinical characteristics for all three groups are summarized in **Table 1**.

**Table 1:** Baseline characteristics of participants.

	DKD ( n=26 )	DM ( n= 8 )	DKD+NDRD( n=6 )	P-value
Age, years	59.00 (55.00-65.75)	59.00 (55.00-61.25)	56.50 (53.75-63.75)	0.736
Gender, n (%)				0.744
Male	21 (80.77%)	6 (75.00%)	4 (66.67%)	
Female	5 (19.23%)	2 (25.00%)	2 (33.33%)	
DM duration, years	12.00 (8.25-18.25)	13.00 (12.00-14.75)	9.00 (4.50-12.00)	0.467
FBG, mmol/L	6.96 (6.01-7.65)	7.04 (7.01-8.81)	5.65 (5.01-6.72)	0.366
HbA1C, %	7.26 ± 1.57	7.17 ± 0.76	6.53 ± 0.55	0.495
Body mass index, kg/m2	24.30 (23.32-26.40)	25.50 (24.80-27.02)	24.20 (23.53-24.20)	0.512
Serum creatinine, umol/L	131.00 (97.80-162.30)	78.00 (56.00-146.00)	83.50 (69.75-116.75)	0.291
24-hour urinary protein, g	2.26 (1.05-3.54)	2.37 (0.11-3.30)	3.75 (3.05-4.51)	0.408
eGFR, ml/min*1.73m2	57.02 ± 27.09	86.26 ± 26.74	65.90 ± 35.15	0.048
SBP, mmHg	140.50 (130.50-151.50)	141.00 (133.75-142.00)	135.00 (126.25-152.00)	0.513
DBP, mmHg	79.38 ± 10.78	82.00 ± 6.74	85.50 ± 14.69	0.438

### 3.2 Taxonomic annotation analysis

**Figure 1:**
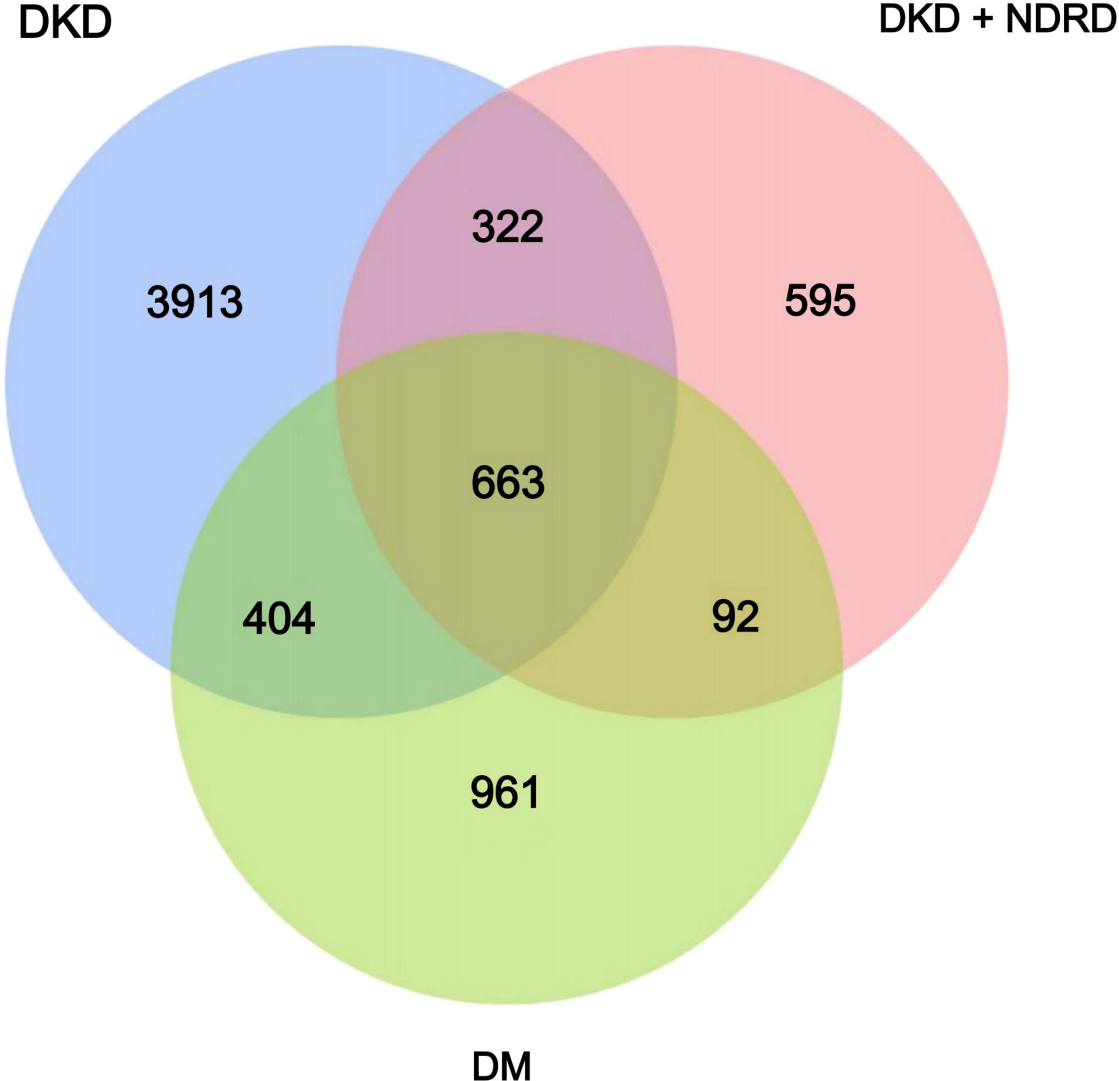
Venn diagram of the DKD, DM, and DKD+NDRD groups. Each circle represents one group; overlapping areas indicate shared ASVs, while non-overlapping areas represent group-specific ASVs.

The Venn diagram visually illustrates the shared and unique ASVs among the DKD, DM, and DKD+NDRD groups, with 663 ASVs common to all groups, 3913 ASVs unique to DKD, 961 unique to DM, and 595 unique to DKD+NDRD. This distribution pattern suggests distinct gut microbial compositions across the three groups, providing preliminary evidence of microbiota divergence. These findings are depicted in **Figure 1**.

### 3.3 Microbial diversity analysis

#### 3.3.1 α - diversity analysis

**Figure 2:**
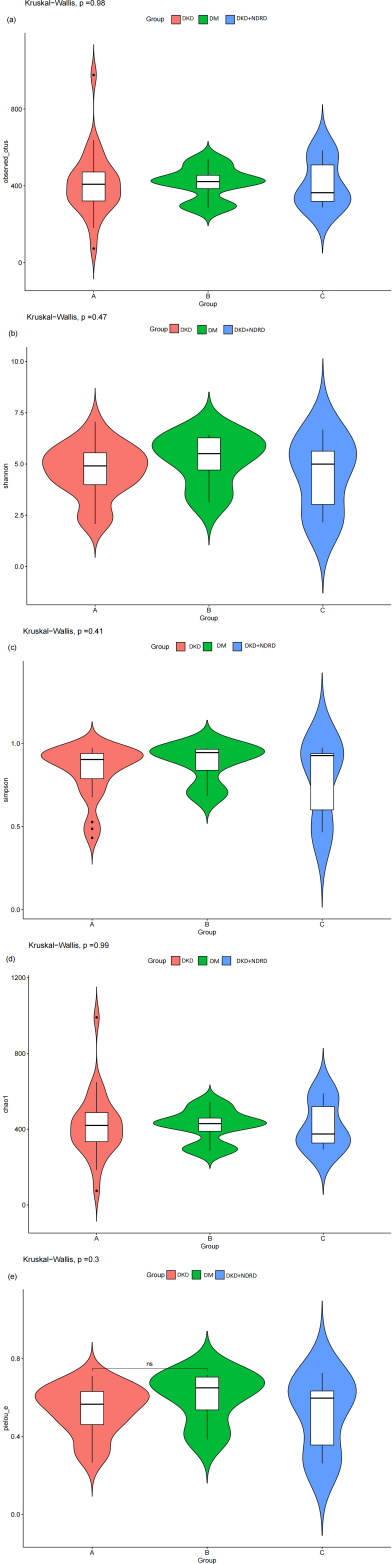
Violin plots of α-diversity indices: **(a)** observed_otus; **(b)** Shannon index; **(c)** Simpson index; **(d)** Chao1 index; **(e)** Pielou’s evenness.

α-diversity analysis reflects species richness and evenness within a single sample. Multiple α-diversity metrics—including Chao1, Observed OTUs, Pielou’s evenness, Shannon, and Simpson indices—were used to compare microbial diversity across the three groups. Group-wise results for each index are shown in **Figure 2**. Statistical analysis using the Kruskal–Wallis test yielded the following p-values: Chao1 = 0.99, Observed OTUs = 0.98, Shannon = 0.47, Simpson = 0.41, and Pielou = 0.3, indicating no statistically significant differences in α-diversity among the groups. These findings suggest that the overall species richness and evenness of the gut microbiota are similar across DKD, DM, and DKD+NDRD groups.

#### 3.3.2 β - diversity analysis

**Figure 3:**
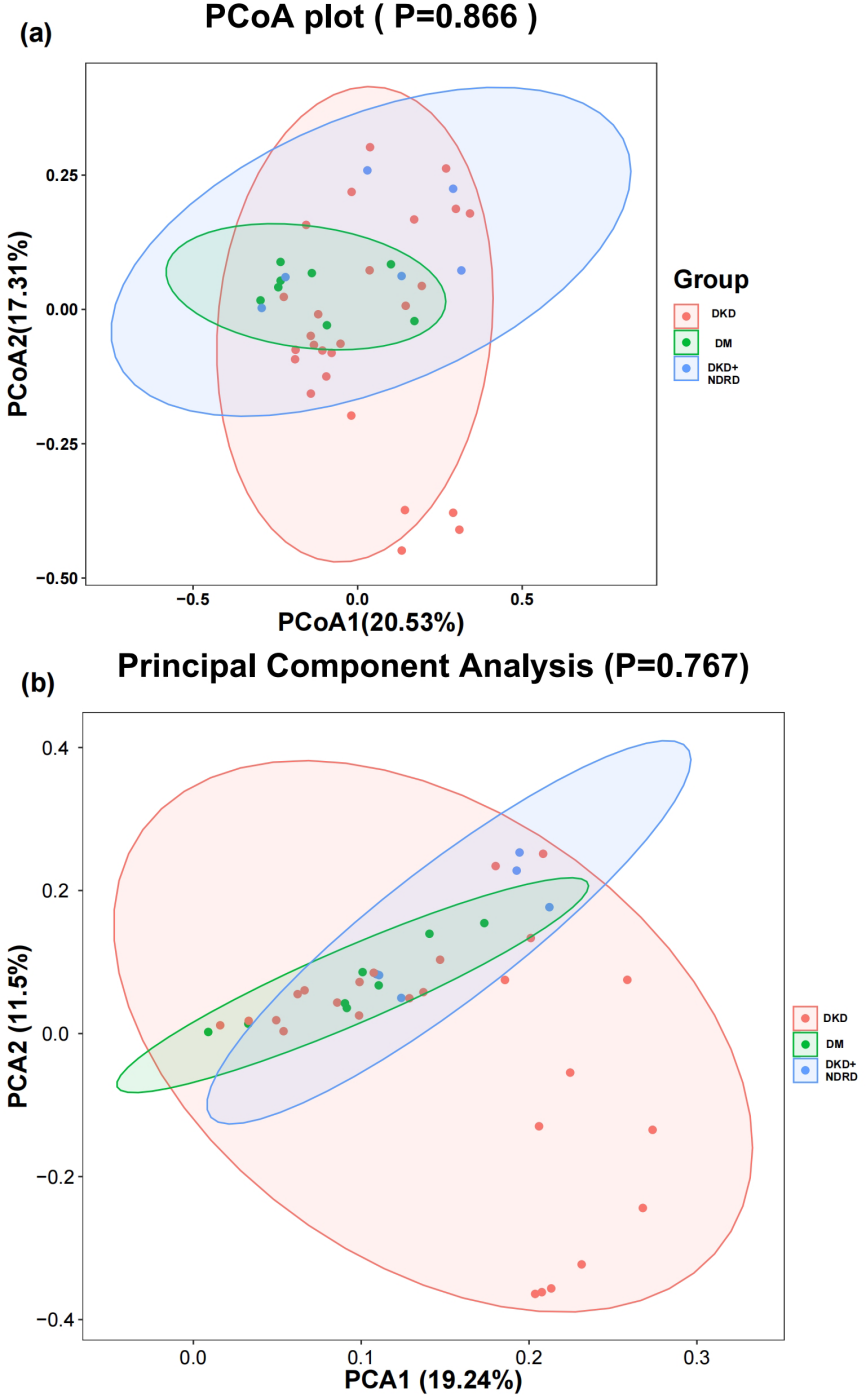
β-diversity analysis: **(a)** PCoA plot; **(b)** PCA plot.

β-diversity analysis reveals intergroup variation in gut microbiota composition. PCoA and PCA were performed using weighted UniFrac and Bray–Curtis distance matrices to visualize community differences. The first two PCoA axes explained 20.53% and 17.31% of the variation, respectively, while PCA1 and PCA2 explained 19.24% and 11.5%. Statistical testing showed no significant differences in β-diversity among the groups (PCoA p = 0.866; PCA p = 0.767). Consistent with this, the visual overlap of samples across the three groups suggests no distinct clustering based on disease status (**Figure 3**).

#### 3.3.3 Microbiota composition

**Figure 4:**
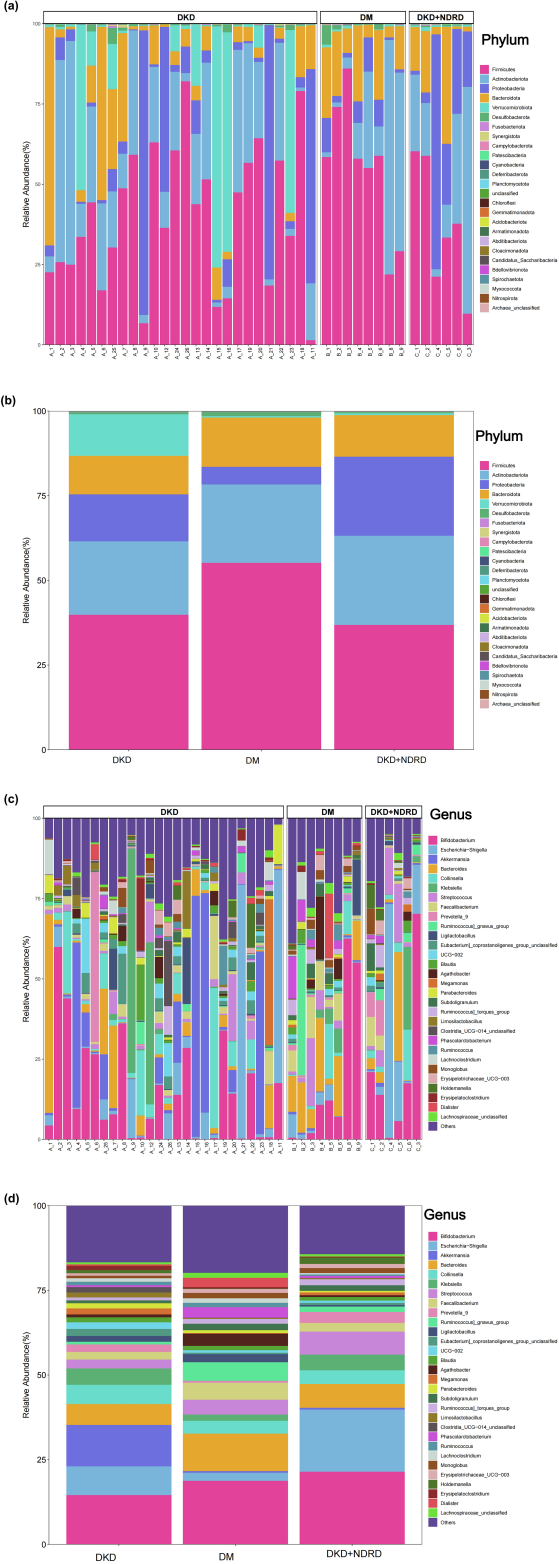
Microbial composition: **(a)** phylum-level composition by sample; **(b)** phylum-level composition by group; **(c)** genus-level composition by sample; **(d)** genus-level composition by group.

Taxonomic profiles were generated at all taxonomic levels to analyze group-specific differences. Stacked bar charts illustrating the top 30 most abundant taxa in each group were used for visualization. Substantial inter-individual variability in microbial composition was observed. At the phylum level, the predominant taxa included Firmicutes, Actinobacteriota, Proteobacteria, Bacteroidota, Verrucomicrobiota, and Desulfobacterota (**Figure 4**).

#### 3.3.4 Differential microbial analysis via LEfSe

**Figure 5:**
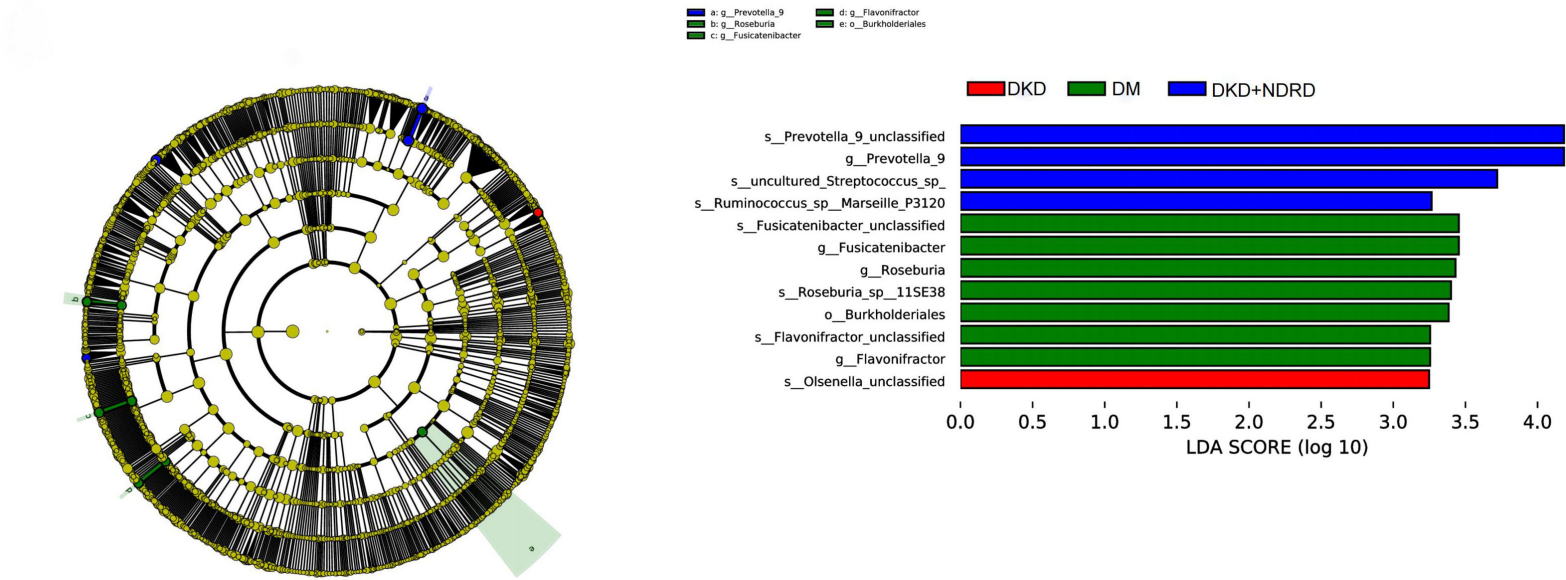
LEfSe analysis results.

LEfSe analysis was used to identify differences in microbial abundance among the DKD, DM, and DKD+NDRD groups. Taxa with an LDA score > 3 and p < 0.05 were considered significantly different, and results were visualized using both a cladogram and an LDA score bar plot. LEfSe emphasizes both statistical significance and biological relevance. The LDA bar plot revealed several taxa with significant differences among the three groups. Specifically, the DKD group was characterized by enrichment of Olsenella, while the DM group showed enrichment of seven taxa including Roseburia, Fusicatenibacter, and Flavonifractor. The DKD+NDRD group was marked by four distinct taxa, including Prevotella_9, Ruminococcus, and Streptococcus. The cladogram on the left further illustrates the phylogenetic relationships of these differentially enriched taxa across the three groups (**Figure 5**).

#### 3.3.5 Functional prediction

**Figure 6:**
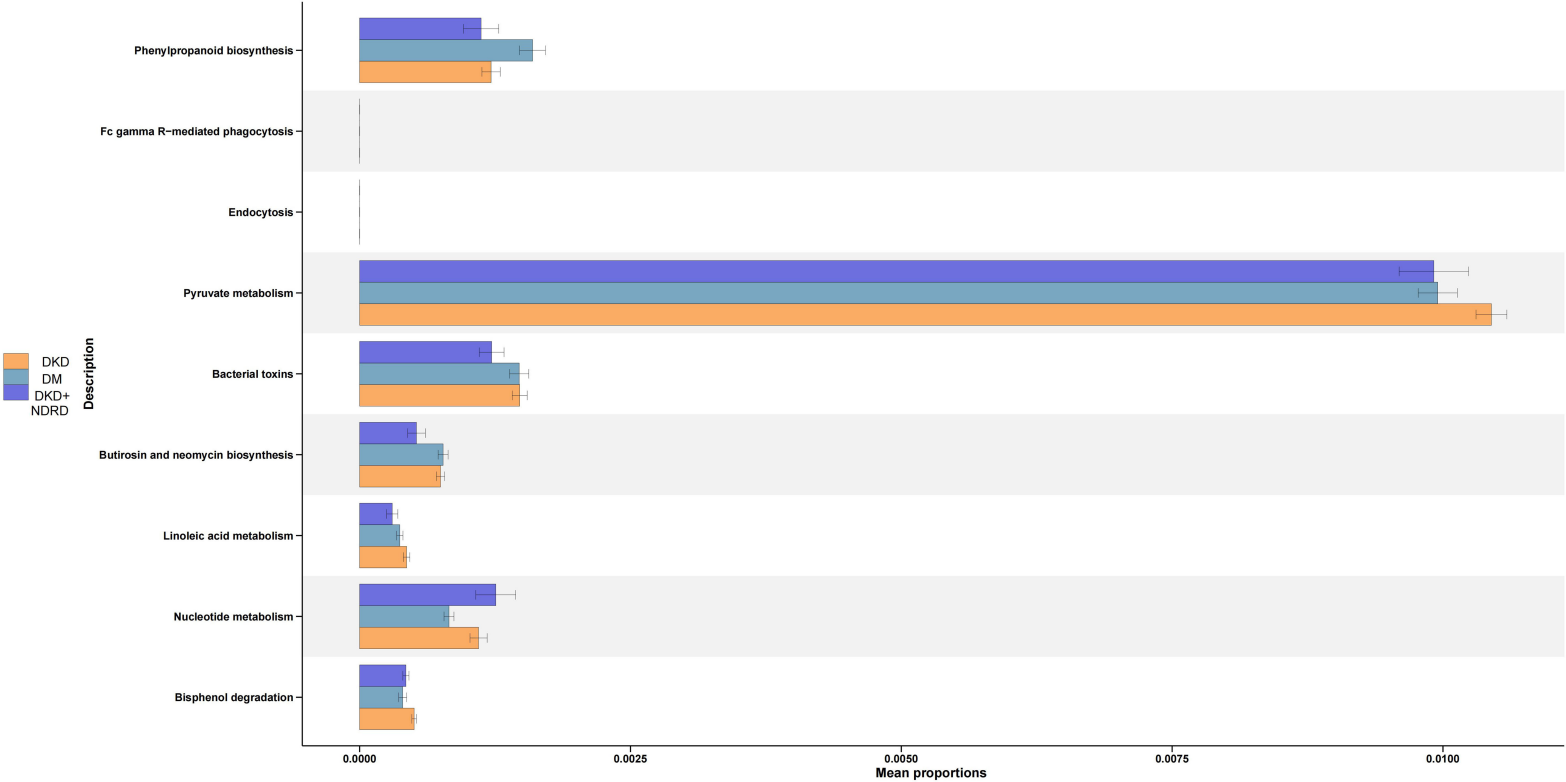
Predicted functional pathway analysis.

PICRUSt2 was used to infer microbial functional potential among the DKD, DM, and DKD+NDRD groups based on 16S amplicon data. The analysis identified distinct microbial functional pathway shifts across the groups. Compared to the DKD+NDRD group, the DKD group showed functional increases in: Phenylpropanoid biosynthesis, Pyruvate metabolism, Bacterial toxin production, Biosynthesis of butirosin and neomycin, Linoleic acid metabolism, and Bisphenol degradation. In contrast, the DKD group showed reduced microbial function in: Nucleotide metabolism. Relative to the DM group, the DKD group demonstrated elevated functions in: Pyruvate metabolism, Bacterial toxin production, Linoleic acid metabolism, Nucleotide metabolism, and Bisphenol degradation. However, the DKD group exhibited reduced function in: Phenylpropanoid biosynthesis (**Figure 6**).

## 4 Discussion

DKD is a major contributor to ESRD. Despite being driven by hyperglycemia and hypertension, tight blood glucose control alone has proven insufficient to halt DKD progression to ESRD or prevent mortality (Dounousi et al., 2015; Lobel et al., 2020). While numerous studies have investigated gut microbiota in DM, research focusing on its role in DKD progression remains limited. Lu X et al. found that Flavonifractor was significantly more enriched in DKD patients compared to healthy controls and DM subjects (Lu et al., 2023). Lecamwasam et al. reported no significant microbial differences between the early and late stages of DM-related CKD (Lecamwasam et al., 2020). In contrast, Tao et al. identified differences in gut microbiota between early-stage DKD patients and DM patients, particularly with respect to Prevotella_9 (Tao et al., 2019).

However, systematic studies on microbiota-targeted interventions to slow DKD progression are still lacking. It is noteworthy that targeting the gut microbiota via probiotic interventions represents a promising new approach for managing diverse disease pathologies (De Angelis et al., 2021). In patients with CKD, probiotics are intended to diminish the levels of uremic organic waste products elevated during disease progression. Synbiotics may exert beneficial effects on CKD-related dysbiosis by shifting the Firmicutes/Bacteroidetes toward a more balanced profile (Vacca et al., 2023). Among CKD patients experiencing malnutrition or protein-energy wasting (PEW), particularly those undergoing hemodialysis, probiotics may synergize with oral nutritional supplements (ONS) components such as extra virgin olive oil, prebiotic fibers, and omega-3 fatty acids, as well as the Mediterranean diet, to alleviate inflammatory and oxidative stress. This multifactorial approach integrates nutritional and microbial therapies, potentially enhancing clinical outcomes (Hevilla et al., 2023). A comprehensive meta-analysis of 21 trials provided additional evidence that probiotics and synbiotics improve renal function and inflammation markers in CKD (Liu et al., 2024). One plausible mechanism is that probiotics help restore gut barrier integrity and suppress the generation of uremic toxins to some extent (Beker et al., 2022; Favero et al., 2022). As gut bacterial populations shift, probiotics help modulate inflammation by restoring equilibrium between inflammatory and anti-inflammatory cytokines (Wang et al., 2020). Moreover, gut-derived metabolites play key roles in maintaining intestinal equilibrium by fermenting amino acids and fibers, synthesizing vitamins and neurotransmitters, and modifying bile acids, thus promoting host well-being (Feng et al., 2019). These functions underscore the systemic impact of the gut microbiome beyond the gastrointestinal tract. Probiotics are likewise beneficial in peritoneal dialysis (PD) populations. Evidence indicates that PD can enhance beneficial microbial populations and decrease pathogenic taxa and uremic toxins, fostering gut microbial balance. Most importantly, through gut microbiota modulation, probiotics can aid in lowering peritonitis risk, maintaining residual kidney function (RKF), controlling inflammation, improving nutrition, and boosting quality of life in PD patients (Stepanova, 2024). Increasingly, studies suggest that traditional Chinese medicine (TCM) and related phytochemicals protect kidney function by regulating gut microbiota and their metabolic outputs through the gut–kidney axis. The renoprotective effects are largely mediated by changes in microbiota composition—specifically targeting Akkermansia, Lactobacillus, and Bacteroides, and modulating the Firmicutes/Bacteroidetes balance. Such modulation enhances SCFA synthesis, lowers uremic toxin accumulation, and helps maintain gut integrity, while mitigating inflammatory and oxidative responses (Zhao et al., 2024). The drugs with the highest use rates in TCM formulations include Poria, Dioscoreae Rhizoma, Glycyrrhizae Radix Et Rhizoma, and F. lycii. Importantly, these TCMs treat CKD by restoring gut microbiota, improving intestinal metabolites, and repairing the intestinal barrier (Li et al., 2023). Poria is reported to reduce proteinuria and renal fibrosis in models of DKD and nephrotic syndrome, partly by suppressing ECM buildup and regulating podocyte injury markers like nephrin and podocin (Guo et al., 2024). Collectively, these insights support the notion that microbiota-targeted approaches—including probiotics, synbiotics, and TCM—may offer a universal therapeutic strategy across diverse CKD etiologies.

Nevertheless, existing findings provide limited insights into microbial differences across diverse DKD-related phenotypes. Based on these prior findings, our study provides a unique profile of the gut microbiota and its functional shifts among DKD, long-standing DM without kidney disease, and DKD+NDRD patients. While neither α-diversity nor β-diversity showed significant differences among the groups, we observed substantial variations in microbiota structure and predicted function. These results imply that certain microbial profiles are closely associated with distinct clinical states and may influence disease trajectories. Although no significant differences in beta-diversity were detected among the groups (PCoA p = 0.866; PCA p = 0.767), this may be attributed to high inter-individual variability, which can obscure subtle but biologically relevant compositional shifts. In contrast, LEfSe analysis identified specific taxa with significant differential abundance across groups. This apparent discrepancy underscores the importance of integrating multiple analytical approaches: whereas beta-diversity captures global community structure, LEfSe can reveal fine-scale taxonomic changes that may serve as potential microbial biomarkers for disease phenotypes.

The DKD group was characterized by elevated levels of Olsenella and reduced levels of Faecalibacterium prausnitzii. Faecalibacterium prausnitzii is a recognized SCFA-producing bacterium (Lv et al., 2021). SCFAs play a crucial role in host health, acting as fuel for gut epithelial cells, reinforcing mucosal integrity, attenuating inflammation, and enhancing peristalsis (Larsen et al., 2010). A decrease in Faecalibacterium prausnitzii abundance may reduce SCFA production, compromising barrier integrity, impairing immune homeostasis, and exacerbating systemic inflammation. Numerous studies have linked low SCFA levels to the progression of diabetes and CKD (Cigarran Guldris et al., 2017; Salazar et al., 2020). This suggests that DKD patients may experience microbial metabolic dysfunctions that exacerbate kidney injury. Functional prediction analysis further supports this hypothesis, showing significant enrichment of pyruvate metabolism and bacterial toxin pathways in the DKD group. However, it is important to acknowledge the limitations of PICRUSt2-based predictions. These inferences are generated based on 16S rRNA gene sequences mapped to reference genomes, and thus do not represent direct measurements of functional gene expression or metabolite activity. As such, they should be interpreted with caution. Validation through complementary approaches—such as metagenomic sequencing or metabolomic profiling—is essential to confirm whether these predicted functional shifts truly reflect *in vivo* microbial activity. Future studies integrating these methodologies will be critical to verifying the role of pathways like pyruvate metabolism and bacterial toxin production in DKD. Pyruvate is a key metabolic intermediate linking glycolysis to the TCA cycle, mainly converted to acetyl-CoA via pyruvate dehydrogenase (PDH) and entering mitochondrial oxidative phosphorylation to generate ATP (Stacpoole and McCall, 2023). Abnormal pyruvate metabolism has been documented in chronic diseases such as heart failure, COPD, and diabetes, and is associated with impaired energy supply and mitochondrial dysfunction (Gray et al., 2014). Given CKD’s shared metabolic features, the enhanced pyruvate metabolism in DKD may reflect metabolic reprogramming under hyperglycemia and hypoxia, possibly indicating impaired mitochondrial oxidation (Forbes and Thorburn, 2018). The increase in bacterial toxins may contribute to CKD-related inflammation, promoting tubular injury and fibrosis (Raj et al., 2009; McIntyre et al., 2011; Feroze et al., 2012). Collectively, these findings suggest that gut microbiota dysbiosis in DKD may drive chronic inflammation and disease progression via metabolic reprogramming and toxin accumulation.

In contrast, the DM group with long-standing diabetes but no kidney disease showed enrichment of beneficial SCFA-producing bacteria such as Roseburia, Fusicatenibacter, and Flavonifractor. This finding is consistent with previous reports showing that DKD patients exhibit increased levels of opportunistic pathogens (e.g., Proteobacteria) and decreased beneficial SCFA-producing bacteria compared to healthy individuals or DM-only patients (Wang et al., 2022). For instance, a meta-analysis revealed that probiotic genera such as Roseburia, Prevotella, and Bifidobacterium were significantly lower in DKD patients than in healthy controls (Wang et al., 2022). In our study, the DM group (long-standing diabetes without kidney damage) had higher levels of Roseburia, while the DKD group showed increased Olsenella and reduced Roseburia. This trend is consistent with Lu et al., who found increased Flavonifractor and decreased Roseburia in biopsy-confirmed DKD patients compared to long-term diabetic patients without kidney disease (Yu et al., 2024). Collectively, these findings suggest that DKD progression is accompanied by a characteristic dysbiosis—loss of beneficial microbes and overgrowth of potential pathogens—which represents a microbial signature of DKD.

However, the coexistence of DKD with immune-mediated renal diseases (NDRD) appears to shift the gut microbiota away from the classical DKD profile. Although Prevotella is typically reduced in DKD patients (Wang et al., 2022), our study identified a marked increase of Prevotella_9 in the DKD+NDRD group, suggesting it as a potential biomarker. Similar findings have been observed in previous microbiota studies of non-diabetic kidney diseases. For example, studies of IgA nephropathy—a typical NDRD subtype—have reported increased abundance of Prevotella-related genera (e.g., Paraprevotella) and correlations with clinical parameters (Dong et al., 2020). In a study by Dong et al., Prevotella levels were positively correlated with serum albumin in IgA nephropathy patients, possibly reflecting better nutritional status or reduced disease activity (Dong et al., 2020). This suggests that Prevotella might exert compensatory or protective effects in kidney diseases characterized by chronic inflammation.

PICRUSt2 analysis further corroborated these findings, showing that pyruvate metabolism and bacterial toxin pathways were enriched in the DKD group, while the DM group exhibited higher predicted activity in linoleic acid metabolism and phenylpropanoid biosynthesis. These results imply a potentially reduced capacity for endotoxin production and distinct metabolic patterns in the DKD+NDRD group. The enrichment of Prevotella_9 suggests enhanced microbial fermentation of carbohydrates, which may influence host metabolic and immune homeostasis. Bacteria of the genus Prevotella are proficient at fermenting dietary fiber into SCFAs, which help maintain glucose balance and enhance insulin sensitivity in the host (Dong et al., 2020).

On the other hand, cell wall components of Prevotella can activate pattern recognition receptors, primarily TLR2, inducing antigen-presenting cells to secrete cytokines such as IL-23 and IL-1, thereby triggering excessive mucosal Th17 responses (Larsen, 2017). This Th17-mediated mucosal inflammation is not confined to the gut, but may also influence systemic inflammation through the translocation of pro-inflammatory mediators and bacterial metabolites (Larsen, 2017). Therefore, the substantial overgrowth of Prevotella_9 observed in the DKD+NDRD group may, on the one hand, enhance gut fermentation capacity and potentially support metabolic homeostasis, while on the other hand, promote chronic inflammation through mucosal immune activation. This dual mechanism provides important insights into how the gut microbiota may contribute to disease progression in patients with DKD complicated by NDRD. While our findings demonstrate associations between specific microbial taxa (e.g., Prevotella_9) and distinct clinical phenotypes, causal relationships cannot be established due to the observational nature of the study. To elucidate the functional relevance of these taxa in DKD progression, future mechanistic investigations are warranted. These may include fecal microbiota transplantation (FMT) into germ-free or antibiotic-treated mice, as well as *in vitro* assays examining the effects of microbially derived metabolites on immune and renal cell responses. Such studies would help clarify whether the observed microbial alterations are drivers or merely consequences of disease progression.

The functional predictions across groups suggest that the spectrum of microbial metabolites may differ under various kidney disease states. This indicates that dysbiosis may directly contribute to disease progression via the gut–kidney axis. The gut microbiota alterations observed in the DKD+NDRD group may have potential clinical applications. Firstly, alterations in characteristic taxa such as Prevotella_9 could serve as biomarkers for differential diagnosis or risk assessment. Currently, diagnosis of DKD combined with other renal diseases relies on renal biopsy, but our findings suggest that gut microbial features (e.g., Prevotella_9 enrichment) might serve as non-invasive indicators of renal pathology. If future large-scale studies confirm consistently elevated fecal Prevotella levels in DKD+NDRD patients, stool microbiota testing could aid in identifying diabetic patients with concurrent kidney pathology. Similarly, the overall degree of gut dysbiosis or specific microbial indices may help predict the risk of DKD progression. Previous studies have also supported the use of gut microbiota as specific markers for chronic kidney diseases: microbiota signatures in IgA nephropathy and membranous nephropathy have been shown to aid in diagnosis and disease activity assessment (Dong et al., 2020). In the context of DKD, some researchers have proposed that changes in specific microbial taxa are closely related to disease development and may serve as potential therapeutic targets (Wang et al., 2022). Our study provides new evidence to support the concept of “microbial biomarkers.”

However, this study has several limitations. We acknowledge that the relatively small sample size—particularly in the DKD+NDRD group (n=6)—may limit the statistical power of our findings and increase the risk of false-negative results. Although a *post hoc* power analysis was not feasible due to the complexity of microbiome data, future studies with larger, multicenter cohorts are warranted to validate the observed microbial signatures and improve generalizability. Importantly, all fecal samples were collected on the day of renal biopsy, prior to the initiation of any additional treatment, to minimize therapeutic interference and capture the native gut microbiota status at the time of pathological diagnosis. Given the invasive nature of renal biopsy and the clinical constraints involved, obtaining same-day stool specimens posed significant logistical challenges—particularly for the DKD+NDRD group, a relatively rare and pathologically heterogeneous phenotype—which partly accounts for the limited sample size. Additionally, the limited cohort size precluded the application of multivariate statistical models such as PERMANOVA or multivariable regression. Although we performed alpha- and beta-diversity analyses and LEfSe biomarker identification, more comprehensive modeling will be feasible in future large-scale studies. Furthermore, although participants with recent antibiotic or probiotic use were excluded, other potential confounding factors—such as dietary habits, smoking status, alcohol consumption, and concomitant medications (e.g., metformin, SGLT2 inhibitors)—were not controlled in this study. These factors may influence gut microbiota composition and contribute to inter-individual variability. Future studies should incorporate these covariates into multivariate analyses to improve the accuracy and interpretability of microbiome–phenotype associations.

Second, the use of 16S rRNA sequencing limits taxonomic resolution to the genus level, preventing strain-level identification. The observed increase in Prevotella_9 may encompass multiple species or strains with distinct functions, and the exact contributors remain unknown. Additionally, functional prediction based on 16S sequencing via PICRUSt2 lacks direct validation from metagenomic or metabolomic data. Further research utilizing metagenomics and metabolomics is necessary to confirm whether Prevotella_9 contributes to metabolic or immune functions as predicted. Third, this cross-sectional design captures only a single time point during disease progression and cannot establish causality between gut dysbiosis and disease progression. Future mechanistic studies are needed to clarify the role of Prevotella_9 in the gut–kidney axis. For example, fecal transplantation from DKD+NDRD patients into germ-free or antibiotic-treated mice could determine whether their microbiota—including Prevotella_9—induces more severe kidney injury than that from pure DKD patients. Alternatively, the functional effects of Prevotella_9-derived metabolites—such as propionate, succinate, or tryptophan metabolites—on kidney and immune cells (e.g., cytokine production, mesangial cell proliferation, or Th17 differentiation) should be explored *in vitro*. Such studies will provide deeper insight into the microbial contribution to the pathogenesis of mixed-type DKD. In summary, our findings reveal distinct microbial signatures and potential functions in DKD patients with concurrent NDRD. With further validation in larger cohorts and mechanistic studies, these microbial changes may be translated into clinical biomarkers or therapeutic targets for precision management of DKD.

## 5 Conclusions

This study, through a biopsy-confirmed triple cohort design, delineates distinct gut microbiota profiles and predicted functions across DKD, long-term diabetes without nephropathy (DM), and DKD with concurrent NDRD (DKD+NDRD). DKD patients exhibited gut dysbiosis characterized by the loss of SCFA-producing bacteria (e.g., Faecalibacterium prausnitzii) and enrichment of potential pro-inflammatory taxa (e.g., Olsenella), accompanied by elevated microbial functions in pyruvate metabolism and bacterial toxin pathways. In contrast, the DKD+NDRD group displayed unique microbial signatures, notably the enrichment of Prevotella_9, and attenuated endotoxin-related pathways, suggesting a possible compensatory microbial response or immune-metabolic interaction. These microbial signatures, particularly Olsenella, Faecalibacterium prausnitzii, and Prevotella_9, offer promising candidates as early biomarkers and therapeutic targets for DKD. Further validation through larger cohort studies and integrated multi-omics analyses is warranted to clarify the precise role of these microbial changes in DKD pathogenesis and clinical management.

## Data Availability

The datasets presented in this study can be found in online repositories. The names of the repository/repositories and accession number(s) can be found below: https://www.ncbi.nlm.nih.gov/, PRJNA1246967.
